# SNMMI Procedure Standard/EANM Practice Guideline on Pediatric ^18^F-FDG PET/CT for Oncology 1.0

**DOI:** 10.2967/jnumed.120.254110

**Published:** 2021-01

**Authors:** Reza Vali, Adam Alessio, Rene Balza, Lise Borgwardt, Zvi Bar-Sever, Michael Czachowski, Nina Jehanno, Lars Kurch, Neeta Pandit-Taskar, Marguerite Parisi, Arnoldo Piccardo, Victor Seghers, Barry L. Shulkin, Pietro Zucchetta, Ruth Lim

**Affiliations:** 1 Department of Diagnostic Imaging, Hospital for Sick Children, University of Toronto, Toronto, Ontario, Canada; 2 Michigan State University, East Lansing, Michigan; 3 Department of Radiology, Massachusetts General Hospital, Harvard Medical School, Boston, Massachusetts; 4 Department for Clinical Physiology, Nuclear Medicine & PET, Copenhagen University Hospital, Rigshospitalet, Copenhagen, Denmark; 5 Schneider Children's Medical Center, Petach Tikva, Israel; 6 UPMC Children’s Hospital of Pittsburgh, Pittsburgh, Pennsylvania; 7 Department of Nuclear Medicine, Institut Curie, Paris, France; 8 University Hospital Leipzig, Department of Nuclear Medicine, Leipzig, Germany; 9 Memorial Sloan Kettering Cancer Center, New York, New York; 10 University of Washington School of Medicine and Seattle Children’s Hospital, Seattle, Washington; 11 Ente Ospedaliero “Ospedali Galliera,” Genoa, Italy; 12 Texas Children's Hospital, Baylor College of Medicine, Houston, Texas; 13 St. Jude Children’s Research Hospital, Memphis, Tennessee; and; 14 University Hospital, Padova, Italy

## Abstract

The Society of Nuclear Medicine and Molecular Imaging (SNMMI) is an international scientific and professional organization founded in 1954 to promote the science, technology, and practical application of nuclear medicine. The European Association of Nuclear Medicine (EANM) is a professional nonprofit medical association founded in 1985 to facilitate communication worldwide among individuals pursuing clinical and academic excellence in nuclear medicine. SNMMI and EANM members are physicians, technologists, and scientists specializing in the research and practice of nuclear medicine.

The SNMMI and EANM will periodically put forth new standards/guidelines for nuclear medicine practice to help advance the science of nuclear medicine and improve service to patients. Existing standards/guidelines will be reviewed for revision or renewal, as appropriate, on their fifth anniversary or sooner, if indicated. Each standard/guideline, representing a policy statement by the SNMMI/EANM, has undergone a thorough consensus process, entailing extensive review. The SNMMI and EANM recognize that the safe and effective use of diagnostic nuclear medicine imaging requires particular training and skills, as described in each document. These standards/guidelines are educational tools designed to assist practitioners in providing appropriate and effective nuclear medicine care for patients. These guidelines are consensus documents, and are not inflexible rules or requirements of practice. They are not intended, nor should they be used, to establish a legal standard of care. For these reasons and those set forth below, the SNMMI and the EANM cautions against the use of these standards/guidelines in litigation in which the clinical decisions of a practitioner are called into question.

The ultimate judgment regarding the propriety of any specific procedure or course of action must be made by medical professionals taking into account the unique circumstances of each case. Thus, there is no implication that action differing from what is laid out in the standards/guidelines, standing alone, is below standard of care. To the contrary, a conscientious practitioner may responsibly adopt a course of action different from that set forth in the standards/guidelines when, in the reasonable judgment of the practitioner, such course of action is indicated by the condition of the patient, limitations of available resources, or advances in knowledge or technology subsequent to publication of the standards/guidelines.

The practice of medicine involves not only the science, but also the art of dealing with the prevention, diagnosis, alleviation, and treatment of disease. The variety and complexity of human conditions make it impossible for general guidelines to consistently allow for an accurate diagnosis to be reached or a particular treatment response to be predicted. Therefore, it should be recognized that adherence to these standards/guidelines will not ensure a successful outcome. All that should be expected is that the practitioner follows a reasonable course of action, based on their level of training, the current knowledge, the available resources, and the needs/context of the particular patient being treated.

PET and computerized tomography (CT) have been widely used in oncology. ^18^F-FDG is the most common radiotracer used for PET imaging. The purpose of this document is to provide imaging specialists and clinicians guidelines for recommending, performing, and interpreting ^18^F-FDG PET/CT in pediatric patients in oncology. There is not a high level of evidence for all recommendations suggested in this paper. These recommendations represent the expert opinions of experienced leaders in this field. Further studies are needed to have evidence-based recommendations for the application of ^18^F-FDG PET/CT in pediatric oncology. These recommendations should be viewed in the context of good practice of nuclear medicine and are not intended to be a substitute for national and international legal or regulatory provisions.

## I. INTRODUCTION

 Although pediatric cancer is relatively rare compared with adult cancer, it is the second most common cause of death, after injury, in children and adolescents (1). Leukemia, brain cancer, and lymphoma account for more than half of pediatric cancers, followed by neuroblastoma, soft-tissue sarcomas, Wilms tumors, and bone tumors (1,2). Childhood cancers are usually different from adult cancers in terms of the epidemiology, histologic patterns, clinical behavior, response to therapy and prognosis. Early and accurate diagnosis of childhood malignancy followed by effective treatment improves the outcomes of these patients. Additionally, reducing mortality from childhood cancer entails increased long-term surveillance of late effects or complications. The role of diagnostic imaging in pediatric cancers is paramount, and this role spans diagnosis, staging, therapeutic evaluation, surveillance, and in some instances, prognostication.

The use of ^18^F-FDG PET/CT as an imaging technique in adult oncology is well established. In many types of pediatric cancers, ^18^F-FDG PET/CT is being increasingly used for staging, prognosis, selection of the biopsy sites, assessment of response to therapy, radiation planning, and follow-up (3–10). PET is a tomographic imaging technique that provides functional or physiologic information through the injection of a positron-emitting radiopharmaceutical. CT is an imaging modality that provides anatomic information by using an x-ray beam. The development of combined PET/CT scanners provides concurrent anatomic and physiologic information, and PET/CT appears to be more accurate in evaluating oncologic patients than either PET or CT alone in many clinical situations (11,12).

^18^F-FDG is the most commonly used radiopharmaceutical for PET in oncology. ^18^F is a cyclotron-produced radioisotope with a half-life of approximately 110 min. FDG is an analog of glucose, which is taken up by cells via cell membrane glucose transporters and subsequently incorporated into the first step of the physiologic glycolytic pathway. Therefore, the degree of uptake of FDG represents the metabolic activity of cells (13). Other PET radiopharmaceuticals have also been used in pediatric oncology. This procedure guideline pertains only to ^18^F-FDG PET/CT in pediatric oncology. The use of other positron-emitting radiopharmaceuticals, indications of PET/CT in children other than oncology (e.g., epilepsy), brain PET, and the use of integrated PET/MRI are not discussed in this procedure guideline.

## II. GOALS

The goal of this procedure standard is to assist nuclear medicine professionals in recommending, performing, interpreting, and reporting the results of ^18^F-FDG PET/CT in pediatric oncology.

## III. DEFINITIONS

A PET/CT system is an integrated imaging device, equipped with both CT and PET scanners. It is capable of acquiring both PET and CT scans, and the reconstructed PET and CT images are spatially coregistered.
PET/CT fusion is the simultaneous display of coregistered CT and PET images.
The CT component of a PET/CT scan can be acquired with variable parameters (i.e., mAs, kVp, and pitch, with or without contrast) to suit clinical need. For example, a low-dose, low-resolution CT scan for attenuation correction and anatomic localization may be sufficient; or a higher-dose, higher-resolution CT scan can be acquired with or without contrast if greater anatomic detail is required.

## IV. CLINICAL INDICATIONS

Common indications for ^18^F-FDG PET/CT imaging in pediatric oncology include, but are not limited to, the following:

Lymphoma (Hodgkin lymphoma [HL] and non-Hodgkin lymphoma [NHL]): Initial staging, response to therapy (for monitoring response during therapy and also to evaluate response after completion of therapy), detection of residual disease, restaging, planning of radiation therapy, and providing prognostic information (8–10,14,15). Routine surveillance imaging with ^18^F-FDG PET/CT after completion of therapy in patients with lymphoma is not recommended (16,17).
Sarcoma (osteosarcoma, Ewing sarcoma, rhabdomyosarcoma, and other soft-tissue sarcomas): Initial staging, response to therapy in osteosarcoma, rhabdomyosarcoma, and Ewing sarcoma, providing prognostic information, and potentially for restaging and detection of relapse (3,6,18–28).

Less common indications with some evidence for the usefulness of ^18^F-FDG PET/CT include neuroblastoma (in ^123^I-MIBG–negative cases, and preoperative or pretherapy prognostic information) (29–34). ^18^F-FDG PET/CT may also be useful in the evaluation of central nervous system tumors (grading, evaluation of response to therapy, prognosis, and differentiation of viable tumor tissue versus postradiation changes) (35–37); head and neck cancer including nasopharyngeal cancer (38); Langerhans cell histiocytosis (LCH) (39–41); posttransplant lymphoproliferative disorder (42,43); germ cell tumors (staging and recurrence) (44); Wilms tumor (45,46); thyroid cancer (negative iodine scan with rising serum thyroglobulin level); neurofibromatosis type 1 (when suspecting malignant transformation of neurofibroma) (7,47); thymic neoplasia; and for guiding biopsies, surgical resection, and radiation treatment planning (4). ^18^F-FDG PET/CT may be beneficial in other specific clinical scenarios in children, and the decision to perform this examination should be a multidisciplinary team decision.

## V. QUALIFICATIONS AND RESPONSIBILITIES OF PERSONNEL

### A. Physician

A nuclear medicine physician with appropriate board certification is the preferred person to report or supervise the performance of PET/CT imaging. Where permitted by national and local regulations, a board-certified pediatric radiologist or diagnostic radiologist with the required additional nuclear medicine training could also perform or supervise this procedure. Please also see the Society of Nuclear Medicine Procedure Guidelines for General Imaging (48).

### B. Technologist

PET/CT scans should be performed by a qualified registered/certified nuclear medicine technologist. Please refer to the following documents for further details: Performance Responsibility and Guidelines for Nuclear Medicine Technologists 3.1 and the EANM Benchmark Document Nuclear Medicine Technologists’ Competencies (49,50). Depending on location of practice, additional qualifications may be required for technologists to be allowed to operate the CT component of the scanner.

### C. Medical Physicist

PET/CT scans must adhere to regional, national, and international quality standards, including international dosimetry and radiation precautions for patients and staff alike. A medical physicist is required for optimization of a PET/CT study and to ensure these established standards are met. A medical physicist can help us to ensure adherence to good practice, radiation dose monitoring, and development of algorithms to minimize the radiation burden of the CT. Please refer to the following documents for further details: American College of Radiology, ACR-AAPM technical standard for medical physics performance monitoring of PET/CT imaging equipment, and the European guidelines on medical physics expert (51,52).

## VI. PROCEDURE/SPECIFICATIONS OF THE EXAMINATION

### A. Request

Close collaboration between the nuclear medicine physician/technologist and the referring physician is necessary for a safe, appropriate, and useful study. The purpose of the study (clinical indication), the histopathologic results (if available), any history of previous relevant studies, previous interventions/therapies (biopsy, surgery, chemotherapy, and radiotherapy), history of recent infections or inflammation, medications (e.g., corticosteroid, granulocyte colony-stimulating factor [G-CSF]), recent administration of enteral barium (per oral or per rectum), whether sedation or analgesia is required, and whether a diagnostic CT (with or without contrast) is needed should all be clarified before protocoling the study. If intravenous contrast material is required, history of any previous reaction to contrast agents should be sought, and current kidney function should be examined. The body coverage should be determined by the nuclear medicine physician and personalized for each patient to minimize radiation burden and based on factors including clinical indication, types of tumor, and any known/suspected sites of disease. In general, imaging of skull base to mid-thigh is sufficient for lymphoma cases as it covers most of the lymph nodes, unless there is an evidence for bone/bone marrow involvement (in which case the extremities should be included), or brain involvement (in which case the acquisition starts from the skull vertex). Total body scanning is suggested in certain tumors that carry the possibility of brain or skull involvement or bone/bone marrow involvement, including sarcoma, neuroblastoma, primary brain tumors and LCH (53,54). When choosing the protocol for the CT portion of the scan, the ALARA (As Low As Reasonably Achievable) principle for radiation exposure should be considered. For example, a PET/CT for initial staging of neoplastic disease may require a diagnostic CT whereas follow-up studies may only require a low-dose CT (with the option to add a diagnostic CT limited to regions of concern.

### B. Patient Preparation and Precautions

1. The study should be explained in detail to the patient and parents/caregivers, who have been provided with information delivered both verbally and in writing. The indication and preparation required for the patient should be reviewed. The patient should be able to lie still for approximately 30 min. The height and weight of the patient should be measured on the day of the examination.
2. Patients should be fasting for 4–6 h before ^18^F-FDG injection to decrease serum glucose level and to maintain a low insulin level. Fasting should be continued during the uptake phase. If the patient is not NPO (nothing by mouth) in preparation for general anesthesia or sedation, then drinking only plain, unflavored water is permitted, and also encouraged to increase hydration and excretion of radiotracer. Patients are not allowed to drink soft drinks containing sugar. Glucose-containing intravenous fluids and parenteral nutrition must be discontinued and replaced by saline intravenous fluid. The serum glucose level should be measured before the radiotracer administration. The glucose level should be below 200 mg/dL (11.1 mmol/L); the optimal level is below 140 mg/dL (7.8 mmol/L). If the blood glucose level is found to be more than 200 mg/dL (11.1 mmol/L), the referring physician should be notified and the study should be rescheduled (55–57).
3. The patient should be sitting in a chair or lying on a bed in a quiet, warm room for ^18^F-FDG injection and during the uptake phase. Patients should avoid talking, playing, exercising, or chewing before and after ^18^F-FDG is administered. Using a pacifier is not contraindicated but its use should be noted as it causes physiologic uptake in muscles of the oral cavity. Vigorous exercise should be avoided for 24 h before the examination to avoid increased muscle uptake (58). To minimize the patient’s distress and muscle tension, and for radiation safety reasons, a peripheral intravenous access should be obtained in advance. Anesthetic cream should be used to minimize pain and distraction methods can be used to decrease the patient’s stress (59). If a central line or Porth-a-Cath has to be used for tracer injection (due to difficult peripheral intravenous access), the line should be flushed with a sufficient amount of normal saline solution. A standard intravenous line must also be flushed with normal saline solution before radiotracer injection.
4. For brain imaging, the patient should stay in a quiet and dimly lit room for both ^18^F-FDG injection and during the uptake phase.
5. Patients should empty their bladder immediately before the scan acquisition to minimize activity in the bladder (thus reducing radiation dose and image artifacts) and to ensure the patient is comfortable for the duration of the study. In small children, urination can be stimulated by holding babies/children in an upright position (gravity) or let them walk (movement) before the scan. It is suggested to change the diaper just before the image acquisition.
6. Bladder catheterization is not routinely recommended for ^18^F-FDG PET/CT studies. It may increase infection risk and stress levels for the child (60). In certain cases, however, when tumors or lymph nodes are close to the urinary bladder, catheterization or intravenous furosemide (0.5–1 mg/kg, maximum 40 mg) may be useful if the patient cannot voluntarily empty his/her bladder.
7. ^18^F-FDG uptake in brown fat can interfere with image interpretation. This occurs more frequently in children than adults. Many strategies are available to minimize brown fat uptake. A practical method to reduce brown fat uptake is to avoid cold by maintaining a warm ambient temperature of the room during uptake phase (minimum 24°C or 75°F) and providing a warm blanket (55,61,62). Patients can be asked to avoid cold environments (including excessive indoor air conditioning) for 1–2 d before the study, and to dress warmly before coming to the hospital or nuclear medicine facility on the day of the study. Premedication has also been used to reduce brown fat uptake (63–65). Oral propranolol, intravenous fentanyl, and a moderate dose of oral diazepam have been shown to be effective in reducing brown fat uptake (63,64,66–68). The recommended dose, route and time of administration, and precautions have been provided in Table 1.
8. The CT component of a PET/CT scan can be acquired with variable parameters (i.e., mAs, kVp, and pitch, with or without contrast) to suit clinical need. For example, a low-dose, low-resolution CT scan for attenuation correction and anatomic localization may be sufficient; or a higher-dose, higher-resolution CT can be acquired with or without contrast if greater anatomic detail is required. Whether the CT component of the PET/CT is low-dose CT vs. higher-dose CT; whether it is with versus without contrast; and the body coverage of the higher-dose portion of the CT scan should all be determined during protocoling and considering ALARA principles. History of allergy to iodinated contrast material, and kidney function (e.g., glomerular filtration rate, serum creatinine level), should be documented before administration of iodinated intravenous contrast. If the patient is under general anesthesia/sedation, oral contrast cannot be given unless authorized by the anesthesiologist.
9. Special consideration should be afforded to diabetic patients:
  The referring physician or primary care physician should be consulted if any changes to dosing of insulin or other hypoglycemic medications are required to perform the study.
  In most cases, it is preferable to schedule a diabetic patient for an early morning time slot.
  Before ^18^F-FDG injection, serum glucose level must be checked. If it is found to be greater than 200 mg/dL (11 mmol/L), the study should be cancelled and rescheduled for a future date with a more effective blood glucose management strategy.
  Patients who receive long-acting insulin at bedtime, and no additional insulin the following morning, should be scheduled for an early morning time slot.
  Patients should not take short-acting insulin formulations before or during PET scanning, as insulin-mediated muscle uptake of ^18^F-FDG may limit scan interpretability. In the event that a patient cannot be scheduled for an early morning time slot, the scan can be performed a minimum of 4–6 h after administration of short-acting regular insulin. Patients who receive regular insulin with breakfast in the early morning (by 6:00 am) should be fasted afterward and scheduled for a noon-time examination.
  Patients using continuous insulin infusions/pumps should be scheduled for a morning scan (8:00 am at the latest) and have breakfast after the study. Accordingly, the pump should be kept on the nighttime/basal setting until the study has concluded (55).
  Although type 2 diabetes is more common in adults, it does occur in children. Patients with type 2 diabetes may be treated with oral hypoglycemic medications, insulin administration, or dietary modifications. Most of these patients can be scheduled early in the morning and should be instructed to take their medications after the scan. If the patient is on metformin, it may be held 2–3 d before the study, especially if the patient has or has had a tumor in the abdomen. Metformin may increase ^18^F-FDG uptake in the colon, small skeletal muscles, and liver (70–72). However, withholding metformin dose before the scan may result in hyperglycemia on the day of the scan, increasing the chance that it may need to be rescheduled and this should be checked with the referring physician. If the CT component is to be performed with intravenous contrast, metformin should be held until after the scan per local institutional protocol or the European Society of Urogenital Radiology recommendation should be followed to prevent acute kidney injury (73). Other oral hypoglycemic medications should be continued as prescribed.
10. For females who have reached puberty, the possibility of being pregnant must be considered, and a pregnancy test may be performed according to institutional policy. In case of pregnancy, the study will be deferred until the risks versus benefits of the scan are discussed with the referring physician and with the patient. For pregnant and breast-feeding patients, see the Society of Nuclear Medicine Procedure Guidelines for General Imaging (48).
11. In general, anesthesia should be avoided. If sedation or general anesthesia is necessary, institutional policy should be followed. Patients who are scheduled for a PET/CT with general anesthesia should be NPO for 6–8 h before the study. When general anesthesia is required, it will be administered immediately before image acquisition (please see SNMMI Procedure Standard for Pediatric Sedation in Nuclear Medicine 3.0 (74)).
12. There is no conclusive data describing the optimum time interval between therapy (chemotherapy or radiation therapy) and ^18^F-FDG PET/CT. Some authors suggest a minimum of 10 d after chemotherapy and 2–3 mo after radiation therapy, similar to the intervals suggested for adults. If a shorter time interval is requested for an urgent clinical situation, the PET/CT scan may still be helpful (75).

**TABLE 1:** Premedication Used to Reduce Brown Fat Uptake

Name of medication	Route of administration	Recommended dose	Maximum dose	Time of administration	Comments and precautions
Propranolol (64–67).	Oral	1 mg/kg usually 20–40 mg	40 mg	60–90 min before ^18^F-FDG injection	Recommended for patients older than 10 y
Fentanyl (63,64,66)	Intravenous	1.0 μg/kg for patients less than 25 kg	50 μg	10 min before ^18^F-FDG injection	Should be injected under the supervision and monitoring of a physician or a trained nurse according to the institutional sedation policy
		0.75 μg/kg for patients less than 25 kg			
Diazepam (63,69)	Oral	0.080–0.096 mg/kg	7.5 mg	30–60 min before ^18^F-FDG injection	Especially useful for anxious patients

### C. Radiopharmaceuticals

#### Radiopharmaceutical: ^18^F-FDG

For the preparation of ^18^F-FDG, the radiopharmaceutical must be prepared by a qualified company or by qualified personnel using methods that fulfill regulatory requirements. Radiopharmaceutical quality control is usually performed by the manufacturer.

The administered activity should be the lowest possible dose that will produce diagnostic image quality. This, in part, depends on the acquisition techniques and the sensitivity of the PET/CT scanner (76–79). The administered activity should follow the 2016 Update of the North American Consensus Guidelines for Pediatric Administered Radiopharmaceutical Activities and the 2016 EANM pediatric Dosage Card (80–82). The administered activity of ^18^F-FDG is 3.7–5.2 MBq/kg (0.10–0.14 mCi/kg) for a body PET/CT scan (with a minimum of 26 MBq [0.7 mCi]) and 3.7 MBq/kg (0.10 mCi/kg) for a brain PET/CT scan (with a minimum of 14 MBq [0.37 mCi]) (Table 2). Please see the EANM pediatric Dosage Card and the North American Guidelines for Pediatric Nuclear Medicine for high-quality images at low radiation dose (81,82). The administered dose, however, can be optimized according to the institutional policy and for certain PET systems (please see the section XI. RADIATION SAFETY IN IMAGING, #3.).

**TABLE 2:** Recommending Linear Dosing Guidelines, Critical Organ Dose, and Effective Dose to Healthy Subjects After Administration of ^18^F-FDG (83–85)

Patient	Administered activity MBq/kg (mCi/kg)	Organ receiving the largest radiation dose* mGy/MBq (rad/mCi)	Effective dose* mSv/MBq (rem/mCi)
Newborn		—	0.21 (0.77)^†^
Child (1 y old)	Body: 3.7–5.2 (0.10–0.14) Minimum: 26.0 MBq (0.7 mCi)	Bladder, 0.51 (1.9)	0.080 (0.30)
Child (5 y old)		Bladder, 0.36 (1.3)	0.048 (0.18)
Child (10 y old)		Bladder, 0.24 (0.91)	0.032 (0.13)
Child (15 y old)	Brain: 3.7 (0.10) Minimum: 14 MBq (0.37 mCi)	Bladder, 0.16 (0.60)	0.022 (0.08)
Adult		Bladder, 0.15 (0.49)	0.019 (0.07)

* Reference (*85*).

† Reference (*84*).

The radiation dose to the patient depends on both the administered dose of ^18^F-FDG and the CT dose. Please see Table 2 for the radiation dose to the patient attributable to injected ^18^F-FDG radiotracer. Table 3 shows examples of injected activity and approximate effective dose for administration of ^18^F-FDG for body (not brain) imaging. The radiation dose contributed by the CT component of the study depends on many factors, which are described in “optimization of CT dose” in the protocol section.

**TABLE 3:** Examples of Injected Activity and Approximate Effective Dose for Administration of ^18^F-FDG for Body (Not Brain) Imaging

Patient	Example weight (kg)	Example injected activity range MBq (mCi)	Effective dose (mSv)
Neonate	7	26–36 (0.7–1)	∼6.5
Child (1 y old)	11	41–57 (1.1–1.5)	∼3.9
Child (5 y old)	20	74–104 (2–2.8)	∼4.3
Child (10 y old)	36	133–187 (3.6–5.1)	∼5.1
Child (15 y old)	60	222–312 (6–8.4)	∼5.9
Adult (sex average)	70	259–370 (7–10)	∼6.0

### D. Protocol/Image Acquisition

The protocol and image acquisition depend on many variables, including PET/CT instrumentation (PET/CT scanners, software, etc.) and indication for the scan (i.e., clinical question, type of tumor, etc.). The protocol should also comply with the institutional policy. Therefore, each institution may have its own unique PET/CT protocols. The study typically commences with the localizer radiograph scan, followed by CT and PET emission imaging. Although this is the common order, most PET/CT systems also support PET then CT imaging, which may be preferred in some settings. The data are reconstructed and transferred to the picture archiving and communication system (PACS) for review.

#### CT

The CT component of the PET/CT should be acquired with the same patient positioning as the PET acquisition. The CT technique factors should be set based on the desired goal for each region of the body. In general, the goals for the CT image, from lowest to highest technique, are: CT for attenuation correction only (CTAC); CT for attenuation correction and anatomic localization (CTAC+CTL); and CT for attenuation correction, anatomic localization, and CT-derived additional diagnosis (CTAC+CTL+Dx) (86,87).

Attenuation correction is an essential step to generate accurate PET images and accounts for loss of detected photon pairs from photon scattering and photoelectric effects. Considering that PET attenuation correction does not require high-spatial-resolution, low-noise CT images, a CTAC scan can be acquired at a very low radiation dose. That said, it does require the CT images to be free from biasing artifacts that can result from photon starvation, an effect in the extremely low-dose regimen. In practice, CTAC images can be acquired with some of the lowest technique settings on a system, but should be tested to ensure that photon starvation, streaklike artifacts are not present in images of appropriate-sized objects (88,89).

Anatomic localization of the PET image is a common goal during PET interpretation. The technique and resulting image quality required for this goal should depend on institutional preference. In most practices, the localization examination can be acquired with a much lower radiation dose than a conventional diagnostic CT study. If the intention is to use the CT for additional diagnostic decisions, whether with contrast enhancement or not, then the CT should be acquired with the standard institutional CT protocols.

All of the CT protocols should be tailored for pediatric patients, using weight-based, size-based, or vendor-provided pediatric protocols that automatically adjust for the patient size (dose modulation). Adjusting the techniques based solely on age is not recommended considering the size variations in the pediatric population (90). Table 4 presents some suggested technique levels for different patient weights for body imaging and are for recommendation only. Technique factors should be set by each institution and may vary based on the local preference, technologist experience, scanner hardware, and available reconstruction methods. When preparing CT protocols with the variety of available technique factors (mAs, kVp, pitch, etc.), modern CT systems will report the predicted CTDIvol (computerized tomography dose index volume) and dose length product (DLP) to reference phantoms (16 or 32 cm). This measure represents the total photon flux and is directly proportional to radiation dose to the patient. Depending on the patient’s size, the radiation dose (as summarized by the CTDIvol levels and DLP) can be reduced by decreasing the mAs or kVp and increasing the pitch (87). A qualified medical physicist should be consulted on all pediatric PET and CT protocols. Other factors including appropriate patient positioning and optimizing the field of view, newer PET/CT scanners with advanced instrumentation/software, reducing patient motion (to avoid repeating image acquisition), and appropriate quality control of equipment are all important factors in reducing radiation dose. For a complete review of optimization of radiation dose to the pediatric patient in PET/CT, please refer to the article by Parisi et al. (87).

**TABLE 4:** Approximate Technique Ranges for the 3 Common Types of CT Acquisitions of the Chest+Abdomen+Pelvis Region for PET/CT for Different Size Patients

			CTDIvol (mGy)*		
Weight (kg)	Lateral distance (cm)	Average age (y)	CTAC	CTAC+CTL	CTAC+CTL+Dx
2.5–12.2	7–11	<1	0.6–0.8	1.1–1.5	1.9–2.5
8.1–23.8	12–18	1–5	0.7–1.0	1.4–2.0	2.4–3.4
14.7–45.6	19–23	5–10	0.8–1.3	1.6–2.6	2.7–4.3
25.9–78.3	24–28	10–15	1.1–1.7	2.1–3.4	3.5–5.7
40.5–95.7	29–33	>15	1.6–2.6	3.1–5.3	5.2–8.8

*CTDIvol ranges represent the dose to the CT dose index 32-cm phantom. Older scanners may report body protocols on pediatric patients as the CTDIvol to the 16-cm phantom. To convert the above CTDIvol 32-cm phantom measurements to the 16-cm phantom, multiply these values by 2.

With these different techniques, there are several potential permutations of acquisitions. These techniques are for general information only. Clinical protocols should be confirmed by each institution.

1. PET/CT with CTAC only. This study would acquire the CT for only attenuation correction over the identical scan range as the PET acquisition. In a pediatric population the lowest possible settings should be used to limit radiation burden. With this technique the CT would provide only limited value for anatomic localization/correlation of the PET signal.
2. PET/CT with CTAC+CTL over the identical scan range as PET.
  For both 1 and 2, a separate diagnostic CT may be needed and acquired for a specific part of the body. For example, diagnostic chest CT may be performed separately to evaluate for lung nodules. This separate CT examination will not be used for PET corrections.
3. PET/CT with a low-dose CT for attenuation correction or anatomic localization over a portion of the PET acquisition and a Dx CT over the remaining portion of the PET acquisition. For example, in a patient with lymphoma who has already had a Dx CT scan of the neck and chest, it may not be necessary to repeat the Dx neck and chest CT scan at the time of the PET examination. The study can be obtained with a CTAC+CTL neck and chest scan, followed by a Dx CT scan of the abdomen and pelvis and completed by a CTAC+CTL scan of the extremities. With this protocol, the extra radiation from a repeated Dx CT scan of the neck and chest will be avoided. If the PET/CT scanner software does not support the acquisition of both Dx- and low-dose CT examinations in the same series to permit attenuation correction, the examination may be acquired with a CTAC+CTL of the whole PET acquisition and a separate Dx CT scan for the diagnostic targeted body area (following approach 2 above).
4. PET/CT with a Dx CT of the same coverage (the CT is used both for attenuation correction and for diagnostic interpretation). Dx CT scan may be performed with intravenous or oral contrast. Using intravenous or oral contrast for a Dx CT scan within a PET/CT study can affect the calculated SUVs in tumor and reference tissues (91,92), however, the difference is generally not clinically significant when performing visual-only assessment; it can be used when contrast is clinically indicated and numerically accurate SUV measurements are not required.

#### PET

The PET acquisition starts immediately after the CT acquisition while the patient is in the same position. The recommended uptake time (time between ^18^F-FDG injection and start of the PET acquisition) should be 60 min. In certain cases, a longer uptake time such as 90–120 min (e.g., a suspicious lesion in the abdomen to increase the target-to-background activity or to identify physiologic bowel activity) or dual-time-point imaging (e.g., to differentiate inflammation vs. viable tumor in the monitoring of brain tumor after therapy) may be useful (75,87,93). The duration of the acquisition depends on the administered dose and the sensitivity of the PET scanner and for conventional systems typically operated with acquisition durations on the order of 1–3 min per bed positions. This time may be shorter with newer, more sensitive PET systems. Additionally, acquisition duration may need to be increased if there is a delay in PET acquisition, in lower sensitivity scanners, for low injected activities, and for obese patients. All follow-up PET/CT scans should ideally be performed within the same timing parameters as the baseline scan. When a follow-up scan differs in duration of uptake time, the measured SUVs are not accurately comparable to the prior scan.

The body coverage of the PET acquisition depends on the indication and suspected sites of disease. On one hand, the study should cover all necessary areas of the body with potential tumor involvement for accurate evaluation of extent of disease. On the other hand, certain parts of the body may be excluded to minimize the radiation dose from the low-dose CT for attenuation correction and to reduce time of acquisition (less patient motion and less need for sedation). For baseline PET/CT in lymphoma, a total-body coverage from vertex to toes is preferred. However, for follow-up PET/CT in lymphoma patients with no suspicion for bone/bone marrow or extremity involvement, it is suggested to obtain the images from skull base to mid-thigh. For other tumors with a higher likelihood of extremity involvement, such as LCH, melanoma, sarcoma, and neuroblastoma, total-body coverage from vertex to toes is suggested.

When a tumor is in the pelvic region or adjacent to the bladder, reversing the direction of the PET acquisition (i.e., from toes to skull base, instead of from skull base to toes), insertion of a bladder catheter, or administration of intravenous furosemide may be useful in decreasing artifacts from very intense, excreted radioactivity in the bladder (94).

Arms can be placed in different positions depending on the indication of the study. However, the shoulders often create CT beam-hardening artifacts that result in inaccurate attenuation correction. When the arms are positioned over the head, beam-hardening artifacts may occur in the neck CT images. Similarly, positioning the arms down at the patient’s sides may result in beam hardening in CT images of the upper chest. Having patients link their hands and rest them on the front of their pelvis, or placing the arms at the patient’s sides but slightly elevated off the bed (in an oblique coronal plane, anterior to the torso), may be more comfortable for the patient and minimize CT beam-hardening artifacts in the chest, while not interfering with neck imaging. Alternatively, arms can be placed above the head for PET/CT of the chest and upper abdomen, followed by arms-down PET/CT of the neck only.

The filtered backprojection reconstruction method has been largely replaced by iterative reconstruction techniques. Iterative reconstruction with time-of-flight capabilities will likely improve the image quality and may enable reduced administered radiation dose while maintaining sufficient image quality. In addition, resolution recovery methods that include point spread function modeling may be beneficial, but should be evaluated by each institution for the desired tasks to ensure that they do not result in inaccurate quantitation. The reconstructed images should be displayed in axial, coronal, and sagittal planes as well as the rotating maximum-intensity-projection image (95). The minimum series to display are attenuation-corrected PET images, non–attenuated-corrected PET images, and coregistered CT images with or without fused PET/CT images (94). For all of the above acquisition and reconstruction settings, each local site should evaluate the available options on their imaging system and, based on the local resources (e.g., time available for each patient scan), adjust protocols to ensure that image quality according to those responsible for interpretation is sufficient for all desired diagnostic tasks.

### E. Interpretation

It is essential to check the quality of images and factors that may influence the SUV (e.g., the administered activity, patient’s weight and height) before interpretation of the study. Any focus of ^18^F-FDG uptake that is greater in intensity than the surrounding background activity, and that is not physiologic, should be noted. This should be compared with a corresponding CT scan. Furthermore, the uptake abnormality should be described qualitatively as being mild, moderate, or severe, with semiquantification measured in terms of SUV, or compared with the blood-pool and liver background activity. Conversely, if an abnormality is noted on CT or other anatomic modality, that abnormality should be characterized on PET in terms of its metabolic activity. Both the attenuation-corrected and non–attenuation-corrected PET images should be reviewed. Deauville 5-point scale (Deauville 5PS) can be used in the initial staging and assessment of treatment response in HL and certain types of NHL.

In order to avoid misinterpreting normal findings as abnormal, the interpreter should be aware of the physiologic ^18^F-FDG activity (please see the section of “Sources of Error”). Furthermore, the patient’s clinical history, indication for scanning, technique of the examination, and any interventions/factors that may interfere with accurate interpretation of findings should be reviewed.

#### Pitfalls: More Common in a Pediatric Population

##### 1. Thymus

In children, there is typically mild to moderate homogeneous ^18^F-FDG uptake in the thymus (96–98). This uptake decreases with age as the thymus undergoes fatty infiltration and eventually involutes in adulthood. Usually, during childhood the thymus is quadrilateral in shape, and in adolescence it takes on a triangular shape (98). Further investigation may be warranted in cases in which the thymic uptake is high, focal, and heterogeneous or if there are CT findings suggestive of an abnormality. It is also possible to see an ectopic thymus especially in the cervical region. Thymic size and uptake may increase after chemotherapy due to a phenomenon called thymic rebound.

##### 2. Brown fat

Activated brown fat tissues often show increased ^18^F-FDG uptake, which is typically bilateral and symmetric and can be seen in the neck, supraclavicular regions, axillae, mediastinum, paravertebral regions, and perinephric areas (96,99). The distribution and pattern of uptake, and correlation with CT scan (hypodense fat-attenuation foci), are helpful to differentiate physiologic brown fat uptake from pathologic ^18^F-FDG activity. Please see “Patient Preparation and Precautions,” section B, for the strategies to minimize brown fat uptake.

##### 3. Muscle uptake

Visualization of muscle uptake is not uncommon in children. Laryngeal muscle uptake secondary to patients talking during the uptake phase, uptake in masseter muscles due to chewing or sucking (pacifier), uptake in the diaphragm and intercostal muscles due to crying, and muscle uptake due to spasm or recent use of a specific muscle are some examples of situations that can result in nonpathologic increased uptake in muscles.

##### 4. Waldeyer’s ring

Physiologic increased ^18^F-FDG uptake is seen in Waldeyer’s ring, which is an area of concentrated lymphoid tissues around the naso- and oropharynx, including the adenoids, palatine, and lingual tonsils (100). Further investigation is needed if the uptake is asymmetric or very intense, or in case there is correlative anatomic abnormality on CT. It is also common to see uptake in reactive lymph nodes in children (101).

##### 5. G-CSF effect

Bone marrow often shows a diffuse intense ^18^F-FDG uptake after hematopoietic stimulating drugs such as G-CSF. An interval of 3 wk between administration of the long-acting form of G-CSF and ^18^F-FDG PET/CT is suggested to minimize the increased marrow uptake caused by G-CSF (102). If ^18^F-FDG PET/CT is done in the middle of chemotherapy (interim PET) and the study cannot be postponed, the reduced sensitivity of bone marrow evaluation due to G-CSF effect should be explained in the report.

##### 6. Motion artifact

Due to the time duration of PET acquisition, patient motion often occurs in children. Such motion can interrupt the PET scan, leading to insufficient image quality and potentially necessitating a repeat study (60). The technologist can reduce motion artifact by using positioning aids such as radiolucent pads, sponges, pediatric boards, and strips of tape across the body or forehead. Motion artifacts can also be avoided with entertainment or distraction methods during the acquisition (e.g., movie screen in the ceiling). If the patient moves between the PET and CT imaging, attenuation in a part of the body may be over- or undercorrected, creating erroneously lower or higher ^18^F-FDG activity. Evaluation of nonattenuated corrected images is helpful for discerning motion artifacts from true uptake abnormality.

#### Pitfalls: Similar to an Adult Population

Other sources of errors include visualization of sites of injection (i.e., radiopharmaceutical injection site, intramuscular injections, vaccinations, etc.), activity in the central line or Porth-a-Cath, physiologic activity in the gastrointestinal tract and urinary tract, physiologic activity in the uterus and ovaries (especially during menstruation and ovulation), and posttreatment inflammation (e.g., after biopsy, surgery, radiotherapy, or less commonly chemotherapy) (69,103,104).

Although ^18^F-FDG PET/CT is very sensitive in many malignant diseases, it is nonspecific and may show increased uptake in other conditions. ^18^F-FDG uptake may increase in infectious and inflammatory conditions (e.g., tuberculosis, fungal, viral, and bacterial infections). Benign neoplasm (e.g., adenoma, fibrous cortical defect, plexiform neurofibroma) may also show increased ^18^F-FDG uptake. Some conditions may result in low relative tumor uptake of ^18^F-FDG and create false-negative results including recent radiation or chemotherapy, tumor necrosis or hypoxia, recent high-dose corticosteroid therapy, hyperinsulinemia and hyperglycemia, and certain tumors that are not consistently ^18^F-FDG–avid (e.g., low-grade brain tumors, well-differentiated tumors including thyroid cancer and neuroendocrine tumors, mucinous tumors, and some types of lymphoma) (69). Small lesions (approximately < 1 cm) may be below the size threshold for characterization by PET, resulting in false-negatives.

## VII. DOCUMENTATION/REPORTING

A generic report template is provided in the SNMMI Guideline for General Imaging (http://snmmi.files.cms-plus.com/docs/General_Imaging_Version_6.0.pdf). The report is a communication tool and a legal document. It should be comprehensive and answer the clinical question. The report should be prepared in a timely manner. If there is an important finding requiring immediate attention, a verbal report via direct communication to the referring physician should be performed, before the written report is completed. The date and time of verbal report and the name of medical provider who received and understood this report should be documented in the written report.

It is good clinical practice to provide a structured report. Standardized reporting structure increases the likelihood that all relevant areas are addressed (105). The following elements are suggested for a structured report:

### A. General Information

Study identification.
Clinical information: Diagnosis, indication(s) for the study (staging/follow-up after treatment, relevant treatment or symptom history).
Comparison examinations: Name the prior examinations related to the current examination (including previous ^18^F-FDG PET/CT, CT, MRI, radiography, and ultrasound) and the dates of these examinations. If prior images are not available for direct comparison, but the report from a prior examination is available, state that correlation is made to this report.

### B. Procedure Description and Imaging Protocol

Administered activity and route of administration of ^18^F-FDG.
^18^F-FDG uptake time
Serum glucose level before the radiotracer administration.
If any other intervention has been done for the PET/CT, such as insertion of urinary catheter, any medication used for reducing brown fat uptake, or any procedures or medication used for reducing pain.
Sedation/anesthesia procedures, if any.
Specific protocol for PET including the body coverage (e.g., skull base to mid-thigh).
Specific protocol for CT including low-dose or Dx CT, if any oral or intravenous contrast was used, body coverage of Dx CT if performed.
Any specific reconstruction protocols such as EANM Research Ltd. (EARL) harmonization protocol (106,107).
If there are any problems that may affect the study quality, such as patient motion.

### C. Body of Report

Description of findings: Describe the location, extent, and intensity of any abnormal ^18^F-FDG activity and describe relevant CT morphologic findings related to PET abnormalities on CT images. The intensity of ^18^F-FDG activity can be described as mild, moderate, or intense. It can alternatively be described semiquantitatively as the SUV (usually SUV_max_). The intensity of ^18^F-FDG activity can be also compared with the blood-pool and liver background activity, especially in lymphoma after therapy (108).

If any specific grading systems such as Deauville criteria are used, the scores should be reported.

Any incidental finding on either PET or CT should be reported.

The clinical question should be addressed even if no abnormality is detected in the area related to the clinical question.

If the CT scan was requested and performed as a diagnostic examination, then the CT component of the study may be reported separately from the PET findings. If there is a separate diagnostic CT report, it should be mentioned in the PET/CT report that this is the case.

Comparison: The findings should be compared with the previous relevant studies.
Limitations: The limitations, if any (e.g., previous G-CSF use and limited bone marrow evaluation, patient motion, altered biodistribution of radiotracer, etc.) and how they may affect the results of the study should be reported.

### D. Impression: This includes the overall interpretation of the findings with recommendations (if any), and specifically addresses the reason for the study

Finally, it is important to review the report to make sure there are no typographic errors (e.g., spelling, left/right mismatch), that all components of a structured report are included, and most importantly, that the clinical question is addressed (109).

## VIII. EQUIPMENT SPECIFICATIONS

See the SNMMI procedure guidelines for tumor imaging using ^18^F-FDG PET/CT and ^18^F-FDG PET/CT: EANM procedure guidelines for tumor imaging: version 2.0 (75,95).

## IX. QUALITY CONTROL AND IMPROVEMENT

For quality control of PET see the following:

SNMMI procedure guidelines for tumor imaging using ^18^F-FDG PET/CT and
FDG PET/CT: EANM procedure guidelines for tumor imaging: version 2.0 (75,95).

For quality Control of CT, see the following:

The “Quality Control” sections of the American College of Radiology Practice Guideline for the Performance of CT of the Extracranial Head and Neck in Adults and Children (110)
The American College of Radiology Practice Guideline for the Performance of Pediatric and Adult Thoracic CT (111), and
The American College of Radiology Practice Guideline for the Performance of CT of the Abdomen and CT of the Pelvis (112).

For quality control of other equipment including computers, software, and monitors, see the SNMMI Guideline for General Imaging (48).

## X. SAFETY, INFECTION CONTROL, AND PATIENT EDUCATION CONCERNS

Imaging should follow local safety protocols. See also the SNMMI Guideline for General Imaging and ACR Position Statement on Quality Control and Improvement, Safety, Infection Control and Patient Education (113).

## XI. RADIATION SAFETY IN IMAGING

Please also see Pediatric Radiopharmaceutical Administration: “Follow the new North American Guideline for Pediatric Nuclear Medicine for high-quality images at low radiation dose” (80).

The radiation dose from CT depends on many factors including whether the study is a diagnostic or low-dose CT. Please see the CT section under “Protocol/Image Acquisition” for further details. The effective dose from the CT component can vary approximately between 1 and 10 mSv (in some cases more). The radiation dose from the PET component depends on the administered activity (based on weight) and the patient’s age. The effective dose per MBq of administered activity is higher in younger children than it is in older children. For example, the effective dose per MBq of administered activity is 0.095 mSv in a 1 y old (9.8 kg), 0.050 mSv in a 5 y old (19 kg), and 0.025 mSv in a 15 y old (57 kg) (Table 2). The bladder receives the highest organ dose in all ages of patients; therefore, adequate hydration before and after the study is of utmost importance.

The following points should be considered regarding radiation exposure for patients and others:

Medical personnel, including nuclear medicine physicians, medical physicists, and technologists, have a responsibility to minimize radiation doses to individual patients, to staff, and to members of the public. The radiation exposure to staff arises from preparation of the radiotracer, administration of the activity, waste handling, and also close contact with the patient after injection (114).
The administered dose should be optimized to allow for production of high-quality diagnostic images.
We suggest that the administered dose recommendation in this guideline be followed. However, the administered dose may be reduced when appropriate. The administered dose may be reduced with new PET systems with a wider axial field of view or equipped with more efficient detector systems (e.g., silicon photomultipliers), or in certain examination protocols. In these cases, it is important to ensure that the reduced-dose study results in high-quality diagnostic images.
